# Improved Trends in the Mortality-to-Incidence Ratios for Liver Cancer in Countries with High Development Index and Health Expenditures

**DOI:** 10.3390/healthcare11020159

**Published:** 2023-01-04

**Authors:** Chang-Cheng Su, Brian-Shiian Chen, Hsin-Hung Chen, Wen-Wei Sung, Chi-Chih Wang, Ming-Chang Tsai

**Affiliations:** 1 Division of Gastroenterology and Hepatology, Department of Internal Medicine, Chung Shan Medical University Hospital, Taichung 40201, Taiwan; 2 School of Medicine, Chung Shan Medical University, Taichung 40201, Taiwan; 3 Institute of Medicine, Chung Shan Medical University, Taichung 40201, Taiwan; 4 Department of Urology, Chung Shan Medical University Hospital, Taichung 40201, Taiwan

**Keywords:** liver cancer, mortality, incidence, mortality-to-incidence ratio, expenditure

## Abstract

Primary liver cancer is one of the leading causes of death globally. Liver cancer has a unique geographical distribution, as its etiologies include chronic viral infections and aging. We hypothesize that the human development index (HDI), current health expenditure (CHE) per capita, and CHE-to-gross domestic product ratio (CHE/GDP) influence the incidence, mortality, and mortality-to-incidence ratios (MIRs) of liver cancer worldwide. Data were obtained from the Global Cancer Observatory (GLOBOCAN) database and the World Health Organization. MIRs and the changes in MIR over time (δMIR) were used to evaluate the correlation of expenditures on healthcare and the HDI disparities via Spearman’s rank correlation coefficient. The crude incidence and mortality were significantly associated with HDI, CHE per capita, and CHE/GDP. Specifically, there were significant associations between δMIR and HDI, as well as between δMIR and CHE per capita. However, there were no significant associations between δMIR and CHE/GDP. Evidently, a favorable liver cancer δMIR was not associated with CHE/GDP, although it had a significant association with HDI and CHE per capita. These results are worthy of the attention of public health systems in correlation to improved outcomes in liver cancer.

## 1. Introduction

Primary liver cancer is one of the leading causes of death globally. In 2018, liver cancer was the sixth most commonly diagnosed cancer and the fourth leading oncological cause of death in both sexes, with about 841,000 new cases and 782,000 deaths annually [1]. According to the World Health Organization (WHO), liver cancer can be classified into two major categories: hepatocellular carcinoma (HCC) and intrahepatic cholangiocarcinoma (ICC), accounting for 75–85% and 10–15% of all liver cancer patients, respectively [1,2]. HCC typically occurs in adults between the ages of 44 and 62, and only 0.5–1% of cases are reported in patients younger than 20 years old [3,4,5]. It is the second most common primary liver malignancy in adolescents and most commonly affects children between 10 and 19 years of age [2]. ICC cases are usually diagnosed in patients in their seventies, as the risk increases with age [6]. Though far less common than HCC in terms of prevalence, the number of ICC cases in both genders has been increasing for unknown reasons in recent years [7].

The main risk factors for HCC include chronic hepatitis B virus (HBV) and hepatitis C virus (HCV) infection, accounting for about 44–56% and 20–21% of all identified HCC cases, respectively [8,9]. Additional risk factors include excessive alcohol consumption, smoking, metabolic diseases, and exposure to dietary toxins, such as aflatoxins and aristolochic acid [10,11]. As for ICC, other than HBV and HCV, primary sclerosing cholangitis (PSC), cirrhosis, intrahepatic stone diseases, metabolic abnormalities, and parasitic infections may contribute to cholangiocarcinoma [12,13,14].

The Human Development Index (HDI), which was created by the United Nations Development Program, is a tangible indicator associated with clinical assessment. The HDI is a summary measure of a country’s average level of achievement. Its calculation accounts for the following major dimensions of human development: leading a long and healthy life, receiving education, and having a decent quality of living [15]. CHE per capita is defined as a measurement of the annual expenditure on healthcare for each person and enables comparisons of countries’ medical expenses [16]. GDP is an economic index calculated by quantifying the value of products or services generated in a region within a year [17]. The ratio of CHE to GDP (CHE/GDP) serves as an important indicator of public health, as it signifies the proportion of GDP that CHE accounts for in a country. Comparisons of monetary outcomes may reveal the importance of healthcare and its disparities worldwide [18]. Lastly, the mortality-to-incidence ratio (MIR) reflects the overall performance of the healthcare sector. MIR has already been identified as a standardized indicator for the evaluation of cancer care quality [19,20].

Primary liver cancer has a unique global geographical distribution due to a wide variety of regional risk factors, such as predisposing viral infections, chronic diseases, aging, and other underlying factors attributing to variations in liver cancer prevalence in each region. Therefore, we sought to determine whether HDI, CHE per capita, and CHE/GDP correlate with clinical statistics, including the incidence, mortality, and MIR of liver cancer worldwide. To date, with the exception of HDI, no conclusions on the relationships between the aforementioned indices and liver cancer have been drawn, and no detailed correlations have been fully clarified to date. Therefore, we conducted this study to analyze crude incidence and mortality rates, MIRs, and changes in MIR over time for primary liver cancer.

## 2. Methods

This epidemiological study included patients diagnosed with liver cancer from 2012 to 2018 using the International Classification of Diseases 10th revision (ICD-10) C22 code for analysis. All patient information was acquired from the online Global Cancer Observatory (GLOBOCAN) database maintained by the International Agency for Research on Cancer. This database offers statistics and graphs for 36 types of cancer using data collected from 185 countries [21].

The HDI serves as a measurement for evaluating the extent of national development. In addition to economic status, the index is composed of two crucial factors. The first is education, which is calculated using the years of education received for people older than 25 years or the number of years that students entering school are expected to receive, and the second is health, which is calculated using life expectancy [22,23]. The HDI values from 2012 to 2018 were obtained from the online United Nations Development Program database by the Human Development Report Office, which covers 189 countries or regions worldwide, with values ranging from 0.954 (Norway) to 0.377 (Chad).

Data on health expenditures, including the CHE per capita and CHE/GDP, was collected from an open-source platform of the Global Health Observatory (GHO), an affiliated WHO entity that provides statistics covering a variety of data related to public health and contains data from all 194 member states. The units for the CHE per capita are US dollars and percentages for the presentation of CHE/GDP.

According to the WHO definition, the crude rate (CR) of incidence and mortality for a certain type of cancer is calculated by dividing the sum of new cases and deaths in a period by the total number of people at risk in the population [24]. Considering the effect that aging may have on the mortality rate, the standardization of age was applied to adjust the CR in the study. The World Standard Population is the most popular approach toward the conversion of crude data, in which the outcome is referred to as the age-standardized incidence or mortality rate (ASR), thus mitigating variations in demographic structures across the globe [24]. Both CR and ASR were presented in terms of the number of patients at risk per 100,000 people. The MIR was defined as the ratio of the mortality CR to the incidence CR [25,26,27,28]. The exclusion criteria for the selection of countries for this study included missing data in the WHO statistics (n = 12), missing HDI data (n = 2), inability to calculable MIR (n = 3), MIR outliers (n = 19), and incidence rates of less than 200 per 100,000 people. Consequently, a total of 111 countries were included in the final analysis. The δMIR was defined as the difference between the MIR in 2012 and MIR in 2018 (δMIR = MIR [in 2012] − MIR [in 2018]) [29].

Associations among the MIR, δMIR, and other factors in various countries were estimated using Spearman’s rank correlation coefficient by using SPSS version 15.0 (SPSS, Inc., Chicago, IL, USA). Values of *p* < 0.05 were considered statistically significant. Scatter plots were generated using Microsoft Excel.

## 3. Results

### 3.1. Epidemiology of Liver Cancer by Region

There were 792,031 new cases and 727,661 deaths from 2012 to 2018 (Table 1). In terms of both newly identified cases and deaths, Asia had the highest cumulative rate, with 12.8 new cases per 10,000 people and 11.8 deaths per 100,000 people. On the contrary, Africa had the lowest cumulative rate, with only 4.9 and 4.8 per 100,000 people for new cases and deaths, respectively. For the MIR, Africa ranked highest among all continents (0.98), while North America ranked lowest (0.77).

### 3.2. Epidemiology and Parameters of Development and Health Expenditure with Regard to Liver Cancer in the Selected Countries

In the original data, the HDI of all 111 countries included in this study ranged from 0.377 in Chad to 0.954 in Norway. The per-capita health expenditure ranged from USD 20 in Congo to USD 9818 in Switzerland. The percentage of GDP spent on health ranged from 2.5% in South Sudan to 18.3% in Sierra Leone. In terms of the incidence CRs, Mongolia had the highest rates. Likewise, with regard to the ASRs of incidence and mortality, Mongolia had the highest rates of 90.0 and 71.8 per 100,000 people for incidence and mortality, respectively. After excluding countries with missing data and MIR outliers, Argentina and Russia had the highest δMIRs of 0.18, while Ukraine had the lowest δMIR of −0.17 from 2012 to 2018.

**Figure 1:**
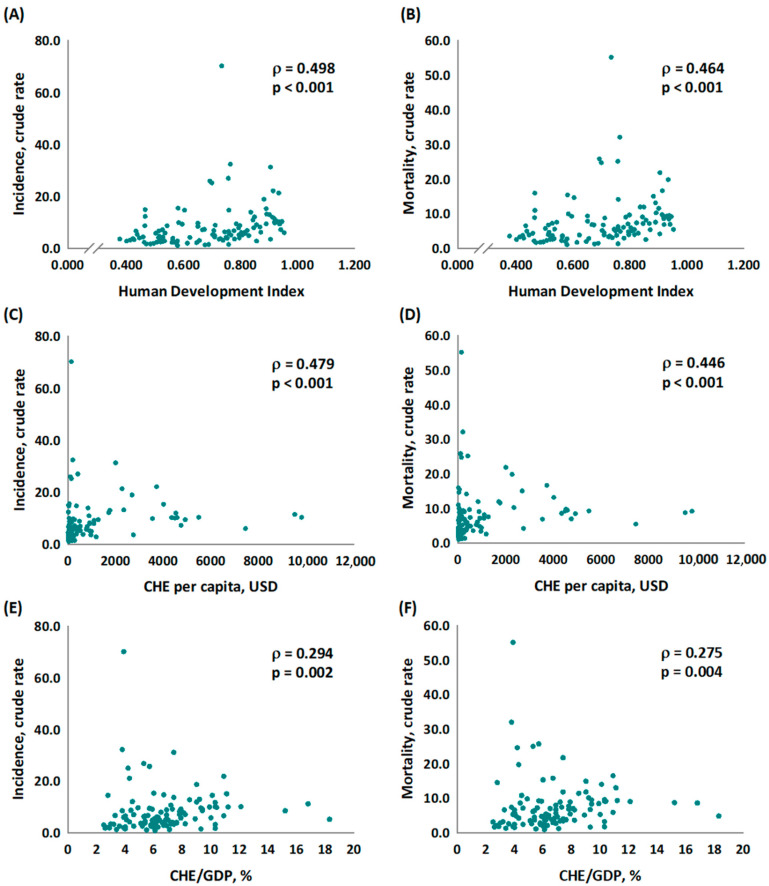
The association between human development index, current health expenditure, and the crude rates of (**A**,**C**,**E**) incidence and (**B**,**D**,**F**) mortality in liver cancer.

We further analyzed the associations between the incidence CR, mortality CR, HDI, CHE per capita, and CHE/GDP. Scatter plots were generated based on the data in Table 2, with countries having more than 923 cases considered eligible for this analysis. A total of 57 countries were included in this phase of analysis. The Pearson correlation coefficients (*ρ*) ranged from 0.498 to 0.275, and HDI showed the strongest correlation with the incidence rate globally. This finding is in line with real-world situations, as detrimental environmental exposures and clinical diagnostic methods are all factors influencing incidence rates. Thus, we noticed that the higher the CRs incidence, the higher the value of HDI, current health expenditure, and CHE/GDP (*p* < 0.001, *p* < 0.001, and *p* = 0.002 in Figure 1A,C,E, respectively). Furthermore, the lower the mortality rates are, the higher HDI, CHE per capita, and CHE/GDP are (*p* < 0.001, *p* < 0.001, *p* = 0.004 Figure 1B,D,F, respectively).

### 3.3. The Association between MIR and δMIR and Parameters of Development and Health Expenditure of the Selected Countries

**Figure 2:**
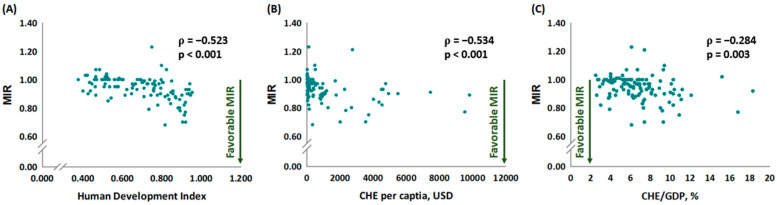
The (**A**) human development index, (**B**) current health expenditure per capita, and (**C**) current health expenditure as a percentage of gross domestic product are significantly associated with the mortality-to-incidence ratio in liver cancer.

**Figure 3:**
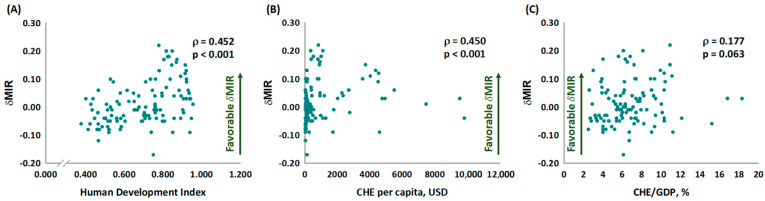
The (**A**) human development index and (**B**) current health expenditure per capita are statistically associated with δMIR in liver cancer. The current health expenditure as a percentage of the gross domestic product (**C**) is not significantly associated with the δMIR in liver cancer.

We analyzed the correlations between MIR or δMIR and HDI, CHE per capita, or CHE/GDP (Figure 2 and Figure 3). The linear regression results showed that MIR was significantly associated with HDI (ρ: −0.523, *p* < 0.001, in Figure 2A), CHE per capita (ρ: −0.534, *p* < 0.001, in Figure 2B), and CHE/GDP (ρ: −0.284, *p* = 0.003, in Figure 2C), suggesting that economic development correlates with overall well-being. Moreover, the results also revealed significant associations between δMIR and both HDI (ρ: 0.452, *p* < 0.001, in Figure 3A) and CHE per capita (ρ: 0.450, *p* < 0.001, in Figure 3B), but not between δMIR and CHE/GDP (ρ: 0.177, *p* = 0.063, in Figure 3C). We confirmed the main result of δMIR and CHE per capita by quintiles CHE per capita into four groups, countries within the third and fourth quintiles had significant high δMIR than those within the first quintile (mean ± standard deviation (SD) of δMIR according quintiles (Q): −0.03 ± 0.05 (Q1, reference); −0.01 ± 0.06 (Q2, p = 0.099); 0.04 ± 0.09 (Q3, *p* = 0.001); 0.05 ± 0.08 (Q4, p < 0.001)). A favorable liver cancer δMIR was still not associated with CHE/GDP while CHE/GDP was divided into quintiles (mean ± SD of δMIR according quintiles (Q): −0.01 ± 0.07 (Q1, reference); 0.011 ± 0.08 (Q2, *p* = 0.677); 0.01 ± 0.07 (Q3, *p* = 0.557); 0.03 ± 0.09 (Q4, *p* = 0.106)).

## 4. Discussion

Our study is the first to consist of a series of assessments for the correlation of liver cancer incidence or mortality with HIR, CHE per capita, or CHE/GDP, with a further analysis of the associations of MIR or δMIR with the same variables by incorporating recent real-world data. The major findings of this study are that both incidence and mortality rates were positively correlated with HDI, CHE per capita, and CHE/GDP. We also noticed that MIR was negatively correlated with HDI, CHE per capita, and CHE/GDP as well. Lastly, we found that δMIR was negatively correlated with HDI and CHE per capita.

Primary liver cancer is unique in its geographical distribution and predisposing viral infection etiologies and can be classified as HCC or ICC. HCC is the most common subtype, with hepatitis B and C being the most important risk factors [1]. Notably, the incidence of HCC generally follows the geographical distribution of the hepatitis B and C viruses [30]. This indicates that regions with HCC risk factors, such as East and Southeast Asia (where HBV is highly endemic), have a higher incidence of primary liver cancer. Our results also show that Asia has the highest cumulative risk of liver cancer.

Currently, the most effective clinical precaution against HBV infection is vaccination. Since its introduction in the 1980s, HBV vaccines have been extensively administered worldwide [21]. A gradual decline in the incidence rate has been observed in East and Southeast Asia, which is indicative of improvements in the quality of life of people in these high-risk areas [31]. Vaccination against HBV has made profound contributions to human society, but the vaccination rate still varies widely among countries [21]. A 2020 report released by the WHO regarding the global coverage of the 3-dose HBV vaccine revealed significant disparities, with vaccination rates as high as 84% in West Pacific areas and as low as 6% in the African region; this underscores the vulnerability of certain countries and the desperate need to provide them with medical assistance to halt the spread of HBV [32]. Russia, which performed 3-dose HBV vaccine coverage 93–97% [33], had the highest liver δMIR while Ukraine, which had only 28–59% 3-dose HBV vaccine coverage [34], had the lowest liver cancer δMIR and highest liver cancer MIR as −0.17 and 1.23, respectively.

Apart from HBV, HCV is also a predisposing factor for liver cancer. Unfortunately, there is still no vaccine available against HCV to date. Preventive measures against transmission may be achieved in socioeconomically developed areas, while regions with poorer medical facilities may fail to contain the disease due to a lack of sterile needles, disinfected surgical equipment, or even curative regimens [21].

Non-viral risk factors, though less predominant than viral infection, may still influence regional distribution. The humid tropical climate in regions such as Southeast Asia serves as a hotbed for parasitic organisms, such as liver flukes. For instance, *Opisthorchis viverini* has been identified as a causative agent of biliary tract cancer, especially ICC; this is particularly true in Thailand, where HCC accounts for only about 30% of all liver cancer cases [31,35]. It is noteworthy that some countries with a historically low rate of liver cancer are seeing a gradual increase in incidence, which may be the result of obesity and diabetes, suggesting the etiological effects of metabolic diseases that are often neglected [24]. In short, the containment of these diseases requires a collective global effort owing to the complexity of liver cancer.

Although treatments for liver cancer have gradually improved the prognosis of patients, primary liver cancer is still one of the leading oncological causes of death [7]. To elucidate the associations between the incidence/mortality of liver cancer and the development of countries, we sought to determine whether HDI, CHE per capita, and CHE/GDP influence the incidence and mortality rates, MIRs, and δMIR of liver cancer worldwide.

Of the 12 scatter plots generated, 11 revealed statistical significance. The only non-significant relationship in the analysis was between δMIR and CHE/GDP (*p* = 0.063). Since the p-value for the analysis of the relationship between δMIR and CHE was below 0.001, we propose that GDP may not be an appropriate indicator for liver cancer. Similar deviations can be observed in the rest of the 11 scatter figures that are statistically significant, in which the p-values of the relationship between incidence or mortality and CHE/GDP had the two lowest values. To our knowledge, one of the shortcomings of GDP is that it does not faithfully recapitulate the prosperity of a nation that has a significant economic gap between the rich and poor. Therefore, all three assessments involving GDP in our study do not exhibit ideal outcomes, implying a poor correlation between liver cancer incidence, mortality, MIR, δMIR, and CHE/GDP values.

Countries with higher development and greater health expenditures have higher incidence rates. The result is possibly due to the widespread and general implementation of early cancer screening systems, facilitating the detection of early-stage liver cancer that is usually asymptomatic [12,36]. Our results showed that the higher the value of HDI, current health expenditure, and CHE/GDP, the higher the CR. It is also worth mentioning that countries with higher HDI, CHE per capita, and CHE/GDP have lower mortality. This implies that well-developed countries have better healthcare systems that allow cancer surveillance and management to be more comprehensive. Patient access to treatments following initial diagnosis may also profoundly affect the prognosis of liver cancer, especially with regard to advanced medical techniques, such as liver transplantation, the Da Vinci Surgical System, and combination therapy in advanced HCC [37,38]. The assumption that socioeconomic factors affect prognosis can be further supported by another finding in the study: MIR, a practical indicator for the evaluation of cancer care, was inversely proportional to HDI, CHE per capita, and CHE/GDP.In highly-developed nations, widespread physical examinations are more likely to take place due to well-organized healthcare infrastructure, experienced medical personal, etc. These qualities allow cancer of early stages to be diagnosed more accurately and health examinations to be carried out more thoroughly among the entire population, benefiting the prognosis of patients with liver cancer in places with higher HDI, CHE per capita and CHE/GDP. Moreover, the number of remedial options are often associated with economic status since developed countries are more likely to conduct elaborate surgeries (e.g., hepatic resection and liver transplantation), obtain medications for target therapies or immunotherapies without financial hardships, or even apply other medical treatments to patients who are not suggested to receive surgeries, such as transarterial chemoembolization (TACE), radiofrequency ablation (RFA) and percutaneous ethanol injection (PEI) with proper equipment and expertise. All the aforementioned factors may either directly or indirectly affect both the incidence and mortality rate of liver cancer. The statistical outcomes presented in our study were an aggregation of huge differences in preventive medicine and clinical practices between developed and non-developed nations, highlighting an unequal chance of having health predominating in the world.

The outcomes of our study in terms of the relationship between liver cancer and HDI are consistent with previous literature, ascertaining the importance of socioeconomic factors in influencing the mortality rate for liver cancer patients [38]. One of the prime results of our study was the identification of CHE per capita as a potential indicator for the epidemiological distribution of cancer, as it exhibited similar results to those of HDI for liver cancer, making CHE per capita another potential proxy for cancer distribution. Before generalizing the potency of CHE per capita in predicting cancer management, further studies are needed to fully examine other types of cancer, especially those with high incidence and mortality rates. Another finding of our study is that there were significant associations between δMIR and HDI as well as CHE per capita. However, the δMIR had no significant correlation with CHE/GDP. This indicates that a country with a higher CHE/GDP does not have a predictable δMIR for liver cancer. The HCC incidence can be decreased after 10–20 years the universal of HBV vaccination and recent cost-effective reference showed HBV vaccine is a good investment strategy in healthcare [39]. Our data showed that Ukraine had the worst liver cancer MIR 2018 and δMIR, while South Korea, provide one of the most detailed hepatitis B screening program [40], had the best liver cancer MIR 2018. The same condition happened in Russia, which had the best liver cancer δMIR between 2012 and 2018. The δMIR of liver cancer did not correlate significantly with CHE/GDP maybe regarded as the cost-effective benefits of HBV vaccination or national HBV/HCV screening program.

There are some limitations in our study. For example, our results were calculated using data from the WHO database, and the accuracy of the primary data would have influenced our secondary database study. Secondly, the use of HDI, CHE per capita, and CHE/GDP to represent healthcare disparities is a major limitation in itself, as the assessment of healthcare quality requires a more discrete and inclusive evaluation beyond these economic indices. Additionally, only data from 57 countries were chosen for further analysis due to the sample size and some MIR outliers in this study. The result may not apply to all sectors of the world and may even deviate from the real-world situation in certain regions. Moreover, we cannot ascertain the proportion of healthcare expenditure allocated to curing or preventing diseases related to liver cancer in each country due to the lack of availability of such data. We could only presume that the increased expenditure on healthcare enhanced the overall conditions for those seeking and receiving medical assistance. Inflation is another confounder that may have an impact on the consistency of the research, as the annual inflation rate is not included in the adjustment of CHE and GDP. An increase in medical spending due to inflation may misguide our interpretation of the quality of medical care, as patients might have only been struggling with the reduced purchasing power of the currency. The staggering prices do not necessarily improve prognoses under certain circumstances, as multiple factors are involved, including, but not limited to, the salaries of medical practitioners, health policies, insurance, and the cost of pharmaceuticals. [41]. Furthermore, our study did not include a subgroup analysis based on gender. Compared to females, males are more likely to use tobacco and consume alcohol, both of which are identified risk factors for liver cancer [11,42]. The generalization of our results does not offer a detailed outcome that fits the respective variations but only a relatively generic indication. Lastly, each risk factor poses different threats and perils via unique etiological mechanisms. The practice of not stratifying the causes may fail to reflect on the genuine relationship between liver cancer and economic values, as the baseline profiles of the study populations were already non-identical. A more discrete subgroup analysis of respective variations should be carried out to rule out potential biases in future studies. However, with a relatively large sample size, our results are still significant and offer novel insights to the medical community regarding the association of liver cancer with economic indices.

In summary, favorable liver cancer δMIRs are not associated with CHE/GDP, but have significant relationships with HDI and CHE per capita. Additionally, CHE per capita is a promising indicator with great potential for liver cancer. This result is worth the attention of public health systems worldwide.

## 5. Conclusions

Our results showed significant associations between δMIR and HDI, as well as between δMIR and CHE per capita. Although a favorable liver cancer δMIR was not associated with CHE/GDP, it had a significant association with HDI and CHE per capita. The correlation between public health systems and outcome of liver cancer is worthy of attention. 

## Figures and Tables

**Table 1:** Summary of the number, crude rate (CR), age-standardized rate (ASR) and mortality-to-incidence ratio (MIR) of liver cancer according to the regions.

	New Cases			Deaths			MIR
Region	Number	CR	ASR	Number	CR	ASR	
Africa	63.186	4.9	8.1	61.962	4.8	8.0	0.98
Asia	578.027	12.8	10.9	533.364	11.8	10.0	0.92
Europe	73.469	10.1	4.9	65.993	9.1	4.1	0.90
Latin America and the Caribbean	34.209	5.3	4.6	32.376	5.0	4.4	0.94
North America	39.416	11.1	6.5	30.714	8.6	4.6	0.77
Oceania	3724	9.2	6.7	3252	8.0	5.5	0.87

**Table 2:** Summary of human development index (HDI), current health expenditure, cancer incidence, cancer mortality, and mortality-to-incidence ratio in liver cancer of selected countries with incidence number > 923, the mean of all countries (n = 55).

	HDI	Current Health Expenditure		Incidence			Mortality			Mortality-to-Incidence Ratio		
Country		Per Capita	% of GDP	Number	CR	ASR	Number	CR	ASR	MIR, 2012	MIR, 2018	dMIR
Argentina	0.830	998	6.8	2074	4.7	3.4	1850	4.2	3.0	1.07	0.89	0.18
Australia	0.938	4934	9.4	2231	9.2	5.5	2020	8.3	4.5	0.93	0.90	0.03
Austria	0.914	4536	10.3	994	11.7	5.2	821	9.6	4.1	0.94	0.82	0.12
Bangladesh	0.614	32	2.6	2940	1.8	2.1	2730	1.6	2.0	0.95	0.89	0.06
Brazil	0.761	780	8.9	11,256	5.4	4.4	10,673	5.1	4.1	1.04	0.94	0.10
Burkina Faso	0.434	33	5.4	1289	6.5	13.6	1262	6.4	13.3	0.97	0.98	−0.01
Côte d’Ivoire	0.516	75	5.4	1110	4.5	8.1	1119	4.5	8.2	0.95	1.00	−0.05
Cambodia	0.581	70	6.0	2474	15.3	20.8	2473	15.3	20.8	0.96	1.00	−0.04
Canada	0.922	4508	10.4	3534	9.8	5.1	3275	9.1	4.2	1.02	0.93	0.09
Chile	0.847	1102	8.1	1387	7.7	5.0	1261	7.0	4.5	1.11	0.91	0.20
China	0.758	426	5.3	378,016	26.8	17.7	352,962	25.0	16.4	0.97	0.93	0.04
Congo	0.459	20	4.3	3519	4.2	7.5	3530	4.2	7.7	0.92	1.00	−0.08
Czechia	0.891	1284	7.3	959	9.2	3.9	772	7.4	3.0	0.76	0.80	−0.04
Egypt	0.700	157	4.2	24,724	25.0	31.2	24,420	24.6	30.8	0.95	0.98	−0.03
Ethiopia	0.470	24	4.0	1574	1.5	2.6	1622	1.5	2.7	0.92	1.00	−0.08
France	0.891	4026	11.1	9525	15.1	7.6	8223	13.0	5.9	0.97	0.86	0.11
Germany	0.939	4592	11.2	7974	10.0	4.1	7467	9.3	3.5	0.84	0.93	−0.09
Ghana	0.596	80	5.9	2681	9.1	14.6	2665	9.1	14.6	0.97	1.00	−0.03
Guatemala	0.651	224	5.7	1627	9.5	13.9	1585	9.2	13.6	0.96	0.97	−0.01
Guinea	0.466	25	4.5	1573	12.1	21.5	1404	10.8	19.2	0.95	0.89	0.06
Hungary	0.845	894	7.2	1012	10.7	5.2	847	8.9	4.2	0.98	0.83	0.15
India	0.647	63	3.9	26,651	2.0	2.1	24,749	1.8	2.0	0.95	0.90	0.05
Indonesia	0.707	112	3.3	17,890	6.7	7.2	17,572	6.6	7.1	0.95	0.99	−0.04
Iran	0.797	366	7.6	3138	3.8	4.2	3085	3.8	4.1	0.95	1.00	−0.05
Italy	0.883	2700	9.0	10,661	18.7	7.6	8490	14.9	5.3	0.86	0.80	0.06
Japan	0.915	3733	10.9	26,641	21.9	6.9	20,048	16.5	4.6	0.90	0.75	0.15
Kazakhstan	0.817	379	3.9	1091	6.0	5.5	974	5.3	4.9	0.95	0.88	0.07
Kenya	0.579	70	5.2	1325	2.6	5.2	1310	2.6	5.2	0.92	1.00	−0.08
Lao	0.604	53	2.8	1008	14.5	21.5	1005	14.5	21.5	0.95	1.00	−0.05
Malaysia	0.804	386	4.0	1869	5.9	6.0	1851	5.8	5.9	1.15	0.98	0.17
Mexico	0.767	535	5.9	6412	4.9	5.0	6044	4.7	4.7	0.95	0.96	−0.01
Mongolia	0.735	152	3.9	2182	70.1	90.0	1716	55.1	71.8	0.89	0.79	0.10
Mozambique	0.446	28	5.4	1165	3.8	6.3	1138	3.7	6.4	0.93	0.97	−0.04
Myanmar	0.584	59	4.9	5214	9.7	9.8	5270	9.8	9.9	0.94	1.01	−0.07
Nigeria	0.534	97	3.6	5053	2.6	4.7	5078	2.6	4.8	0.97	1.00	−0.03
Pakistan	0.560	38	2.7	4280	2.1	3.0	4159	2.1	2.9	0.96	1.00	−0.04
Peru	0.759	323	5.3	2032	6.3	6.0	1967	6.1	5.8	0.98	0.97	0.01
Philippines	0.712	127	4.4	9234	8.7	10.8	9096	8.6	10.6	0.96	0.99	−0.03
Poland	0.872	797	6.3	2252	6.0	3.1	1935	5.2	2.5	1.04	0.87	0.17
Portugal	0.850	1722	9.0	1191	11.9	5.1	1178	11.8	4.9	0.90	0.99	−0.09
Russian Federation	0.824	524	5.6	9513	6.7	3.7	10,282	7.2	3.9	1.25	1.07	0.18
Senegal	0.514	36	4.0	1060	6.5	12.2	1068	6.6	12.3	0.95	1.02	−0.07
Singapore	0.935	2280	4.3	1206	21.1	11.1	1128	19.7	10.2	0.98	0.93	0.05
South Korea	0.906	2013	7.4	15,687	31.1	16.8	10,978	21.7	11.0	0.73	0.70	0.03
Spain	0.893	2354	9.2	5822	13.0	6.2	4518	10.1	4.4	0.82	0.78	0.04
Tanzania	0.528	32	6.1	1483	2.5	4.7	1462	2.5	4.7	1.00	1.00	0.00
Thailand	0.765	217	3.8	22,051	32.2	20.3	21,900	32.0	20.2	0.95	0.99	−0.04
Turkey	0.807	455	4.1	4044	5.0	4.2	3989	4.9	4.1	0.93	0.98	−0.05
Uganda	0.528	46	7.3	1793	4.1	7.4	1534	3.5	6.6	0.95	0.85	0.10
Ukraine	0.750	125	6.1	1714	4.0	2.2	2106	4.9	2.6	1.06	1.23	−0.17
United Kingdom	0.920	4356	9.9	6458	10.0	4.7	5415	8.4	3.6	0.97	0.84	0.13
United States of America	0.920	9536	16.8	35,868	11.2	6.7	27430	8.6	4.7	0.80	0.77	0.03
Uzbekistan	0.710	134	6.2	1352	4.2	5.2	1195	3.7	4.6	0.97	0.88	0.09
Venezuela	0.726	973	3.2	1077	3.3	3.3	1039	3.2	3.2	0.94	0.97	−0.03
Viet Nam	0.693	117	5.7	24,493	25.7	22.9	24,571	25.7	22.9	0.95	1.00	−0.05

## Data Availability

The datasets used and/or analyzed during the current study are publicly available in the Global Cancer Observatory (GLOBOCAN) database (https://gco.iarc.fr/today/, accessed on 28 September 2020), United Nations Development Program/Human Development Report Office (http://hdr.undp.org/en, accessed on 28 September 2020) andWorld Health Statistics database (https://www.who.int/gho/publications/world_health_statistics/en/, accessed on 28 September 2020).
